# Exploring the relationship between glycemic variability and muscle dysfunction in adults with diabetes: A systematic review

**DOI:** 10.1007/s11154-025-09942-z

**Published:** 2025-01-29

**Authors:** Shanshan Lin, Sof Andrikopoulos, Yan-Chuan Shi, David Sibbritt, Wenbo Peng

**Affiliations:** 1 School of Public Health, University of Technology Sydney, Ultimo, NSW 2007 Australia; 2 Australian Diabetes Society, Sydney, NSW 2000 Australia; 3 Neuroendocrinology Group, Garvan Institute of Medical Research, Darlinghurst, NSW 2010 Australia

**Keywords:** Type 2 diabetes, Glycaemic variability, Muscle dysfunction, Sarcopenic obesity, Diabetes complications, Older adults

## Abstract

This review is to systematically explore the relationship between muscle dysfunction and diabetes in adults, and to examine the impact of glycemic variability on muscle health and the development of diabetes-related complications. The review was conducted using three databases: MEDLINE, Scopus, and EMBASE, targeting peer-reviewed journal articles written in English and published from January 2014 to September 2024. The methodological quality assessment of the eligible studies was conducted using Joanna Briggs Institute Critical Appraisal Checklists. A total of 17 studies were included. Most studies were undertaken in Asian countries (*n* = 11) and focused on adults with type 2 diabetes (*n* = 12). There were 8,392 adults with diabetes, and their mean age ranged from 52 to 75 years old. The measurements for muscle function and glycemic variability varied across studies. The research findings regarding the relationship between muscle dysfunction and glycemic variability metrics among adults with diabetes, both with and without complications were inconsistent. For adults with diabetes and sarcopenic obesity, poor glycemic control was identified as an independent risk factor for sarcopenic obesity. Additionally, all included studies were rated as moderate or high quality in relation to their methodology. In conclusion, this review underscores the complex and inconsistent relationship between glycemic variability and muscle dysfunction in older adults with diabetes. Poor glycemic management is a significant risk factor for sarcopenic obesity, highlighting the need for tailored interventions to improve glycemic control and muscle health in this population.

## Introduction

Diabetes is a major public health concern worldwide, particularly among older adults. The prevalence of type 2 diabetes (T2D) increases with age, resulting in a higher burden of disease in the elderly. According to the Centers for Disease Control and Prevention, approximately 29.2% of adults aged 65 and older have diabetes in the period 2017–2020, with T2D being the most common form [1]. Furthermore, in South-East Asia, 1 in 11 adults (90 million) had diabetes and this is predicted to increase by 69% to 152 million by 2045, while 51.2% of adults with diabetes are undiagnosed [2]. Without intervention, approximately one in three with pre-diabetes will develop T2D within 10 years [3].

Obesity is another complex chronic disease often closely linked with many other health conditions, such as diabetes. According to statistics from the World Health Organisation (WHO), 890 million people (16% of adults aged 18 years and over) were living with obesity in 2022 [4]. Additionally, obesity is prevalent in the U.S. such that 46.3% of adults aged 40–59 years were living with obesity and 38.9% of those 60 years and over [5]. The Australian Institute of Health and Wellbeing (AIHW) shows that in 2022 obesity rates in adults over the age of 65 years was 40%, compared with 32% for the whole adult population [6]. The prevalence of obesity in the Asian region is continuously rising. For example, 79 million adults were living with obesity in Southeast Asian countries in 2020 [7] and, based on data from the Chinese Centre for Disease Control and Prevention, the prevalence rates of obesity among adults increased by 13.3% [8]. Thus, the high prevalence of diabetes and obesity underscores the urgent need for effective management strategies to mitigate the associated health risks and economic burden.

The global economic burden of diabetes is projected to increase from $1.3 trillion in 2015 to $2.2 trillion by 2030, even if countries meet international targets for diabetes management [9]. The economic burden of managing both conditions, diabetes and obesity, is substantial. In 2022, the total annual cost of diabetes in the United States was estimated at $412.9 billion, including $306.6 billion in direct medical costs and $106.3 billion in indirect costs such as lost productivity and premature mortality [10]. Older adults with diabetes incur approximately double the per capita annual health care expenditures compared to other age groups, for example, 65–69 years incurred an average cost of $15,354 per person as compared to 25–44 years of $6,532 [10]. Similarly, adults aged 65 years and older with obesity face significantly higher medical costs, with annual expenditures increasing by $1,920 to $4,710 depending on the severity of obesity [11]. It is worth noting that the disparities in healthcare systems between developed and developing countries may impact the overall economic burden of diabetes. Compared to developed countries, developing countries have scarce resources for healthcare (e.g., no universal health coverage, insufficient workforce, and lack of medicine supplies) and challenges to allocate additional resources for diabetes management along with the existing communicable disease issues [12, 13].

Muscle health is crucial for maintaining physical function, metabolic health, and overall quality of life in older adults. Muscle dysfunction is a broad term to describe various conditions related to muscle loss and weakness, including sarcopenia [14, 15]. Sarcopenia, the age-related loss of muscle mass and function, is a significant concern in this demographic. A meta-analysis article reported the pooled prevalence of sarcopenia was 18% among adults with type 2 diabetes, ranging from 6.3% to 47.1% [16]. When combined with obesity, it leads to a condition known as sarcopenic obesity, which further complicates disease management and increases the risk of disability and mortality [17]. Another meta-analysis article demonstrated that the pooled prevalence of sarcopenic obesity was 27% in people with all types of diabetes, with the highest rates in North America and Asia [18].

Furthermore, older adults living with diabetes face unique challenges in managing their condition. Reduced physical activity and muscle strength due to age and diabetes, changes in metabolism, and the presence of multiple comorbidities further complicate disease management [19]. Additionally, older adults often experience greater variability in blood glucose levels, which can impact muscle health and the development of diabetes-related complications [20, 21]. The interplay between muscle dysfunction, glycemic variability, and diabetes-related complications is complex and multifaceted, necessitating a comprehensive understanding to inform effective interventions.

Therefore, this study aims to systematically review the existing literature on the interplay between muscle dysfunction, and diabetes in older adults; investigate how glycemic variability impacts muscle health and the development of diabetes-related complications.

## Methods

This systematic review followed the Preferred Reporting Items for Systematic Reviews and Meta-Analyses (PRISMA) reporting guidelines [22].

### Literature search strategy

A literature search was conducted in electronic databases, including MEDLINE, Scopus, and EMBASE for studies published from January 2014 to September 2024. A similar search strategy was employed to search the databases via a combination of Medical Subject Headings, keywords, and other terms related to diabetes/diabetes complications, glycemic variability, and skeletal muscle dysfunction. The searches were restricted to peer-reviewed journal articles or articles in press written in English. The detailed search strategy can be found in Table 1.

**Table 1:** Search strategy

Diabetes and diabetes complication	Diabetes mellitus [MeSH term & keyword] OR diabet* [Title/Abstract] OR Diabetes complications [MeSH term & keyword] OR Diabetic nephropathies [MeSH term & keyword] OR Diabetic kidney disease [Keyword] OR Diabetic cardiomyopathies [MeSH term & keyword] OR Diabetic heart disease [Keyword] OR Cardiovascular diseases [MeSH term & keyword] OR Heart diseases [MeSH term & keyword] OR Stroke [MeSH term & keyword] OR Heart failure [MeSH term & keyword] OR Renal insufficiency [MeSH term & keyword] OR Kidney failure [Keyword] OR Kidney diseases [MeSH term & keyword] OR Nephropathy [Keyword]
AND	
Glycemic variability	Insulin [MeSH term & keyword] OR Blood glucose [MeSH term & keyword] OR Glycemic variability [Keyword] OR Glycaemic variability [Title/Abstract] OR Glycaemic control [Keyword] OR Glycemic control [Title/Abstract] OR hyperglycaemia [Title/Abstract] OR Hyperglycemia [MeSH term & keyword] OR Hypoglycaemia [Title/Abstract] OR Hypoglycemia [MeSH term & keyword]
AND	
Skeletal muscle dysfunction	Muscular atrophy [MeSH term & keyword] OR Muscle atrophy [Title/Abstract] OR Sarcopenia [MeSH term & keyword] OR Skeletal muscle [MeSH term & keyword] OR Muscle weakness [MeSH term & keyword] OR Muscle wasting [Keyword] OR Muscle mass [Keyword] OR Muscle loss [Keyword]

^*^ truncation for the literature search

### Study selection criteria

Studies were included if they met the following criteria: (1) study participants were adults living with diabetes; (2) muscle-related body composition measurements (e.g., muscle strength and muscle mass) were included in the studies; (3) one of the outcomes was related to glycemic variability (e.g., Glycated hemoglobin A1c (HbA1c) and fasting plasma glucose level); and (4) primary data findings were reported.

Studies were excluded if they (1) were animal, pharmacological, or *in vitro* studies and (2) focused on children or adolescents living with diabetes. Although there was no restriction on study design for this review, some types of articles were not eligible, including literature reviews, research protocols, book chapters, conference abstracts, case reports, and commentaries/editorials. In addition, meta-analyses would be considered only if at least two eligible randomized controlled trial articles were available.

### Data extraction

The articles identified in the search from each database were imported into EndNote 20.2.1 (Clarivate Analysis, Philadelphia, USA) for management. Duplicate articles were removed from the results. Two authors (WP & SL) independently screened the titles and abstracts of articles to identify whether the articles met the inclusion criteria. If the title and abstract of an article did not provide enough information for making the decision, the full text was reviewed for eligibility. When consensus on including/excluding articles was not reached, the other authors were consulted.

The details of the eligible articles were then extracted into a pre-developed table that presents the characteristics of the studies, such as the country of the study, study design, and sample demographics (Table 2). Two authors (WP & SL) completed this table independently and discussions were conducted to resolve any disagreement. The other authors were consulted when consensus was not reached.

**Table 2:** Characteristics of included studies

Author; Country; Publication year	Inclusion/Exclusion criteria of samples	Study design;	Study setting;	Sample size	Sample characteristics (baseline)	Study limitations
Yoon et al.; Korea; 2016	Inclusion criteria: n/a Exclusion criteria: Patients with a history of cerebrovascular accidents. Patients with diabetes mellitus who had glycosylated hemoglobin levels of < 6%	A cross-sectional study (no data reported regarding the recruitment period)	Community-based	People with diabetes: 79	Age (mean): 73 years; Sex: 0% females; Diabetes type: n/a; Diabetes duration: n/a; Probable sarcopenia prevalence: n/a	1) The cross-sectional design limited the interpretation of the causal relationship between glycemic control and muscle quality. 2) The lack of correlation between the perimuscular fat area and muscle quality could be associated with the confounding effect of the subcutaneous fat area. 3) Data was part of the KLoSHA cohort study, which was uneven distribution between control and diabetes groups
Fung et al.; Singapore; 2019	Inclusion criteria: A diagnosis of type 2 diabetes for at least 1 year from the data of their electronic medical records Exclusion criteria: Patients with known risks which hindered or compounded sarcopenia assessment; physical disabilities that affect handgrip and/or walking; using electronic implants and living in residential care facilities	A cross-sectional study between October 2017 and March 2018	Primary care clinic	People with diabetes: 387 (Asian patients)	Age (mean): 68 years; Sex: 47% females; Diabetes type: type 2; Diabetes duration: from 1 to 50 years; Probable sarcopenia prevalence: 27.4%	1) The causal and chronological relationship of the associated factors with sarcopenia cannot be established from this cross-sectional study. 2) The potential recall bias, data reliability and accuracy cannot be objectively ascertained in the self-reported variables. 3) The findings are not generalizable to the wider, heterogeneous population of older patients with type 2 diabetes in Singapore
Kim et al.; Korea; 2019	Inclusion criteria: Newly diagnosed, drug-naïve patients with type 2 diabetes from the Korea Guro Diabetes Program (KGDP) cohort study Exclusion criteria: Patients used metformin	A cross-sectional study between September 2014 and June 2017	N/A	People with diabetes: 233 (Asian patients)	Age (mean): People without low muscle mass and abdominal obesity – 54 years, people with low muscle mass – 56 years, people with abdominal obesity – 53 years, people with both low muscle mass and abdominal obesity – 58 years; Sex: People without low muscle mass and abdominal obesity – 42% females, people with low muscle mass – 45% females, people with abdominal obesity – 52% females, people with both low muscle mass and abdominal obesity – 53% females; Diabetes type: n/a; Diabetes duration: n/a; Probable sarcopenia prevalence: n/a	1) We did not consider muscle function when defining sarcopenia. 2) This study recruited only Asian men and women. 3) Due to the inherent limitations of a cross-sectional study, it was not possible to assess a causal relationship of both low muscle mass and abdominal obesity with metabolic disturbances including insulin resistance and diabetic complications
Ogama et al.; Japan; 2019	Inclusion criteria: (1) Patients with type 2 diabetes treated with antidiabetic agents; (2) aged 65 years or older; (3) living in their houses; (4) with families or caregivers who support self-monitoring of blood glucose; and (5) a Mini-Mental State Examination score > = 10 for cognitive impairment Exclusion criteria: (1) Severe hearing loss and visual impairment; (2) severe health conditions, such as cardiac failure, renal disorder or liver dysfunction; and (3) neurological disorders other than AD or aMCI	A cross-sectional study between 2014 and 2016	Hospital	People with diabetes: 69	Age (mean): 75 years; Sex: 47.8% females; Diabetes type: type 2; Diabetes duration: n/a; Probable sarcopenia prevalence: 11.6%	1) Due to the cross-sectional design, causal relationships should be carefully considered. 2) The sample size was relatively small. 3) The cognitive impairment group was assigned based on the criteria of probable or possible AD and aMCI, but biomarkers for AD pathology were not assessed in this study. 4) We did not use elaborate equipment to evaluate muscle mass and walking speed
He et al.; China; 2020	Inclusion criteria: Patients aged 50 years or older with a previous diagnosis of type 2 diabetes Exclusion criteria: (1) Serious systemic diseases; (2) tuberculosis; (3) severe mental illness; (4) cognitive disability or an inability to record in the diet diary and cooperate with the examination; (5) implantation with metal stent or pacemaker *in vivo*; and (6) current or recent weight loss surgery	A cross-sectional study between January 2016 and March 2018	Hospital	People with diabetes: 1,125	Age (mean): Females—62 years, Males—64 years; Sex: 48% females; Diabetes type: type 2; Diabetes duration (average): 11 years; Probable sarcopenia prevalence: n/a	1) Due to the inherent limitations of the cross-sectional study design, we could not determine a causal relationship between sarcopenia and those correlated metabolic variables. 2) physical activity was not analyzed. 3) We did not analyse some of the key variables stratified by age. 4) This study focused on a study population of Chinese adults with type 2 diabetes
Kataoka et al.; Japan; 2020	Inclusion criteria: n/a Exclusion criteria: Patients had severe cardiac or lung disease, acute or chronic musculoskeletal disorders, acute metabolic dysregulation, neurological or endocrine disorders, a history of stroke, metal implants, a stent and/or pacemaker inserted; Patients had previous or current asymmetric proximal lower-leg weakness and significant limitations in their activities of daily of living	A cross-sectional study between April 2012 and March 2018	Hospital	People with diabetes: 130	Age (mean): 60 years; Sex: 44% females of patients with diabetic polyneuropathy; Diabetes type: type 2; Diabetes duration (mean): 10.8 years; Probable sarcopenia prevalence: n/a	1) The study design was a cross-sectional study, which cannot clearly demonstrate the relationship between muscle mass and DPN. 2) It is unclear whether or not similar results can be obtained in patients with type 1 diabetes. 3) Only the presence or absence of DPN was considered a determinant of muscle mass
Orlando et al.; Italy; 2020	Inclusion criteria: Patients aged 50 to 80 years with diabetes duration > 5 years and had no difficulty in performing the basic activities of daily living Exclusion criteria: n/a	A cross‐sectional study between January 2014 and May 2015	Primary care clinic	People with diabetes: 146 (Caucasian patients)	Age (mean): 67 years; Sex: 45.2% females; Diabetes type: type 2; Diabetes duration (mean): 15 years; Probable sarcopenia prevalence: n/a	1) The lack of a nondiabetic control group. 2) The cross‐sectional design of the study which does not allow an assessment of cause‐effect relationships between muscle fatigability and diabetic complications
Oguz et al.; Turkey; 2021	Inclusion criteria: Patients between the ages of 18–70 and with a body mass index (BMI) of 25–40 kg/m^2^ Exclusion criteria: Patients used non-steroid anti-inflammatory drugs or prednisolone (> 7.5 mg/day) and had contraindications. Patients with type 1 diabetes, renal impairment, renal replacement therapy, pregnancy, infectious diseases, muscular dystrophy, lipodystrophy and cancer, Cushing syndrome, growth hormone, severe vitamin D deficiency, hypogonadism, hypothyroidism, and hyperthyroidism	A cross-sectional study (no data reported regarding the recruitment period)	Primary care clinic	People with diabetes: 90	Age (mean): 55 for people with type 2 diabetes without sarcopenic – 55 years, diabetes with sarcopenic – 54 years; Sex: 78% females; Diabetes type: type 2; Diabetes duration (mean): 11 years; Probable sarcopenia prevalence: 25.6%	1) The sample size was relatively small
Park et al.; Korea; 2021	Inclusion criteria: Patients aged over 30 years and have type 2 diabetes mellitus Exclusion criteria: Patients with malignancy, acute infection, amputated extremities, and those receiving dialysis	A cross-sectional study between November 2017 and March 2019	Hospital	People with diabetes: 1,230	Age (mean): 63 years; Sex: 41.4% females; Diabetes type: type 2; Diabetes duration: n/a Probable sarcopenia prevalence: n/a	1) The study could not confirm a causal link between sarcopenia and CVD because of its cross-sectional design. 2) The results may not be generalizable to all patients with type 2 diabetes mellitus. 3) The dietary intake of the participants was not measured. 4) The statistical power was insufficient to analyse the effect of muscle mass and muscle strength on IS and PAD
Sugimoto et al.; Japan; 2021	Inclusion criteria: Ambulatory patients with type 2 or type 1 diabetes who were aged 40 years or older at recruitment; patients had finished 1-year follow-up measurements of physical performance Exclusion criteria: n/a	A longitudinal cohort study between May 2016 and December 2017	Hospital and primary care clinic	People with diabetes: 588	Age (mean): 70 years; Sex: 41% females; Diabetes type: type 2 or type 1; Diabetes duration: n/a; Probable sarcopenia prevalence: 6.3%	1) Relatively small participant number, and short follow-up duration. 2) We did not consider the nutritional status, the dosages of antihyperglycemic agents and insulin. 3) We did not evaluate the level of physical activity
Yano et al.; Japan; 2021	Inclusion criteria: Patients with a diagnosis of heart failure according to the Japanese Circulation Society/Japanese Heart Failure Society Guidelines Exclusion criteria: Patients with chronic kidney disease stage IV and V, patients receiving concurrent treatment with glucocorticoids, patients with valvular heart diseases who were scheduled for surgical procedures, and patients with type 1 diabetes	A cross-sectional study between January 2016 and May 2019	Hospital	People with diabetes: 70	Age (mean): 73 years; Sex: 31% females; Diabetes type: type 2; Diabetes duration: n/a; Probable sarcopenia prevalence: n/a	1) Due to the cross-sectional design with a small number of patients in a single center, there might have been selection bias in the study subjects. 2) The study findings may not be extrapolated to ambulatory patients with heart failure. 3) Plasma angiotensin II level was not measured in the present study. 4) Comparative analyses of the effect of diabetes on muscle mass between heart failure patients and age-matched non-heart failure controls were not performed
Hiromine et al.; Japan; 2022	Inclusion criteria: Patients with type 1 or type 2 diabetes who were aged ≥ 40 years at recruitment Exclusion criteria: n/a	A cross-sectional study between May 2016 and December 2017	Hospital and primary care clinic	People with diabetes: 812	Age (mean): People with type 1 diabetes – 63 years, People with type 2 diabetes – 70 years; Sex: People with type 1 diabetes – 58% females, People with type 2 diabetes – 40% females; Diabetes type: type 1 (n = 57), type 2 (n = 755); Diabetes duration (mean): People with type 1 diabetes – 19 years, People with type 2 diabetes – 16 years; Probable sarcopenia prevalence: n/a	1) We could not infer causality because of the cross-sectional study design. 2) We could not examine the associations between type 1/type 2 diabetes and sarcopenia and its components due to the small number of patients with type 1 diabetes. 3) We estimated the ASM using bioelectrical impedance analysis devices, and did not use dual-energy X-ray absorptiometry
Sencan et al.; Turkey; 2022	Inclusion criteria: n/a Exclusion criteria: Patients without type 2 diabetes; the presence of a cardiac pacemaker, infection, malignancy, chronic inflammatory disease, dementia, acute cerebrovascular disease	A longitudinal cohort study between September 2016 and July 2019	Hospital	People with diabetes: 92	Age (mean): 73 years; Sex: 64% females; Diabetes type: type 2; Diabetes duration (mean): n/a; Probable sarcopenia prevalence: 27.7%	1) Retrospective design. 2) Using an indirect method (BIA) to measure muscle mass. 3) Patients' daily calorie and protein intake were not evaluated in detail in this study
Shi et al.; China; 2022	Inclusion criteria: Patients aged 35 years or older with type 2 diabetes which was defined according to the WHO definition Exclusion criteria: Patients with serious health conditions, cognitive disability, or an inability to cooperate with the examination; Patients who were pregnant or contemplating pregnancy	A cross-sectional study between 2017 and 2019	Hospital	People with diabetes: 1,084	Age (mean): Females—57 years, Males—52 years; Sex: 39.9% females; Diabetes type: type 2; Diabetes duration (mean): Females—9 years, Males – 8 years; Probable sarcopenia prevalence: n/a	1) Due to the limitation of observational studies, we could not identify a causal relationship between low muscle mass and glucose fluctuations. 2) Some detailed information which may impact glucose control was not available in this study. 3) Standard capillary blood glucose monitoring was applied to evaluate glucose levels, while CGM might represent a more accurate glucose profile. 4) The majority of participants were Chinese
Terada et al.; USA; 2022	Inclusion criteria: Adults with type 2 diabetes with a body mass index ≥ 25 kg/m^2^ and between the ages of 45–76 years Exclusion criteria: Patients underwent DXA	A secondary data analysis of the Look AHEAD trial between August 2001 and April 2004	Primary care clinic	People with diabetes: 1,369	Age (mean): Females—58 years, Males who were in the high-FMI group—60 years, Males who were in the low-FMI group—61 years; Sex: 62.7% females; Diabetes type: type 2; Diabetes duration (mean): Females – 7 years, Males who were in the high-FMI group—8 years, Males who were in the low-FMI group – 7 years; Probable sarcopenia prevalence: n/a	1) The data did not include muscle functions. 2) There is a large heterogeneity in methods for determining fat mass and muscle mass
Herder et al.; Germany; 2024	Inclusion criteria: Patients with known diabetes duration of less than 1 year from the German Diabetes Study Exclusion criteria: n/a	A cross-sectional study (no data reported regarding the recruitment period)	N/A	People with diabetes: 842	Age: n/a; Sex: n/a; Diabetes type: n/a; Diabetes duration (year): ≤ 1 year; Probable sarcopenia prevalence: n/a	1) The cross-sectional design. 2) Data for skeletal muscle strength or quality and physical performance were not available. 3) The smaller sample size and the unavailability of biomarker measurements for glucose-tolerant people in the GDS. 4) Study participants were mainly of European descent
Mooshage et al.; Germany; 2024	Inclusion criteria: n/a Exclusion criteria: (1) Patients who received insulin therapy. (2)pPatients were age < 18, pregnant, an estimated glomerular filtration rate < 60 mL/min, and any contraindications for magnetic resonance imaging or administration of MRI contrast agents. (3) patients with a history of spine surgery or disc extrusion, any risk factors for sarcopenia or neuropathy other than diabetes and any chronic neurological diseases	A cross-sectional study between June 2018 and March 2020	Hospital	People with diabetes: 46	Age (mean): 64 years; Sex: 74% females; Diabetes type: type 2; Diabetes duration (mean): 7.2 years; Probable sarcopenia prevalence: n/a	1) The sample size does not allow for ruling out all potential demographic confounders. 2) The cross-sectional nature of the study does not allow for drawing definite conclusions on the predictive value of the association between DCE-MRM parameters and fasting state insulin levels. 3) The small cohort of healthy controls. 4) Only fasting insulin levels and the HOMA index were employed to assess insulin resistance. 5) The lack of histological data on the microcirculation of the skeletal musculature

### Study quality assessment

The methodological quality of the eligible studies was assessed by the Joanna Briggs Institute (JBI) Critical Appraisal Checklists, including the checklist for cross-sectional studies and the checklist for cohort studies. The tool for cross-sectional studies consists of eight items covering the assessment of the sample selection criteria, the setting description, the measurement of exposure, etc. The tool for cohort studies is comprised of 11 items, including the assessment of the compatibility of the exposed and unexposed groups from the same population, follow-up time, strategies used to deal with confounders, etc. There are four options for those items (i.e., yes, no, unclear, and not applicable). The full checklists can be found at https://jbi.global/critical-appraisal-tools.

In line with previous research [23, 24], a score was assigned to the answer to each appraisal checklist question to indicate the quality of the methodology: Yes = 1; Unclear = 0.5; No = 0; NA = Not Applicable. The total scores of eligible studies can thus be calculated by adding the score of each item [25]. That is, for the checklist for cross-sectional studies, scores from 0 to 4 indicate low quality; those from 5 to 6 indicate moderate quality; and those from 7 to 8 indicate high quality. Regarding the checklist for cohort studies, scores from 0 to 5 indicate low quality; those from 6 to 8 indicate moderate quality, and those from 9 to 11 indicate high quality. Two authors (WP & SL) independently assessed the quality level of the included studies. Discussions were conducted for any discrepancies with other authors.

## Results

**Fig. 1:**
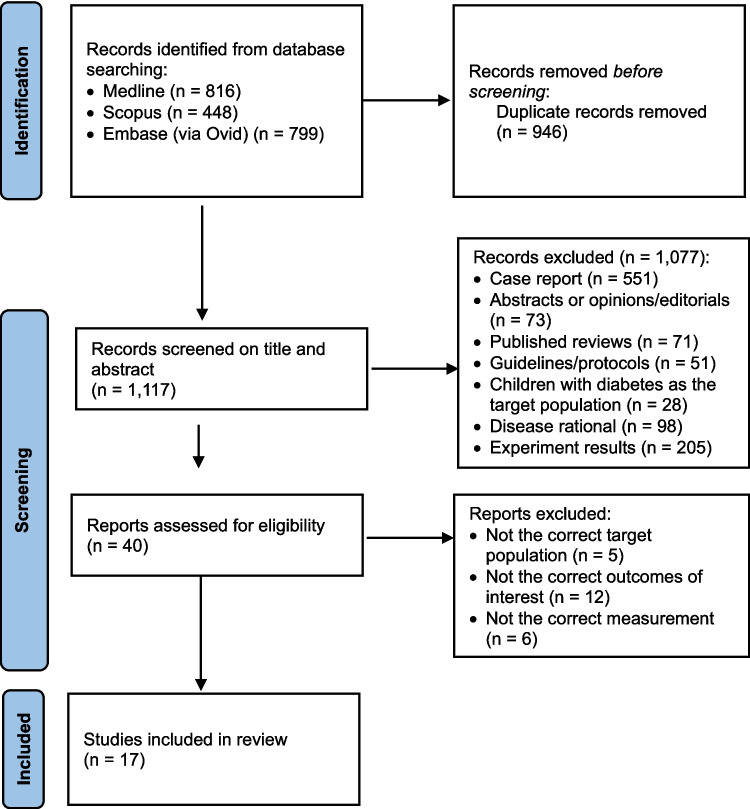
PRISMA flow diagram for the identification of studies

Figure 1 illustrates the study selection process and results via the PRISMA flow diagram. There were 2,063 articles identified in the initial literature search, which were used to screen for eligibility. After the removal of 946 duplicates, 1,077 articles were excluded according to the exclusion criteria above. The most common reasons for exclusion were case reports and animal/*in vitro* studies. The full text of the remaining 40 articles was reviewed and 23 of them were excluded based on the inclusion criteria. The primary reasons for exclusion were inappropriate target population, irrelevant outcomes, or unsuitable measurements. As such, a total of 17 articles (i.e., 17 studies) met the inclusion criteria and were included in this review [26–42].

### Quality assessment of included studies

Results of the methodological quality assessment for cross-sectional studies were shown in Table 3 and those for cohort studies were presented in Table 4. The 14 cross-sectional studies were scored between 5.5 and 8 (out of 8) and were rated as moderate or high quality. The two cohort studies were scored as 8.5 and 9 (out of 11) and were rated as moderate or high quality. That is, all studies included in this review had a low risk of bias. It is worth noting that all studies, including both cross-sectional and cohort studies, reported the outcomes in a valid and reliable way and used appropriate statistical analysis methods. One study [40], which reported the secondary data analysis of a cohort study, was not assessed for its methodology quality as that study did not report results based on primary data.

**Table 3:** Quality assessment of the methodologies of included cross-sectional studies

	Item 1	Item 2	Item 3	Item 4	Item 5	Item 6	Item 7	Item 8	Total score	Level of quality
Yoon et al.; 2016	Yes	No	Yes	Yes	Yes	Not clear	Yes	Yes	6.5	Moderate quality
Fung et al.; 2019	Yes	Yes	Yes	Yes	Yes	Not clear	Yes	Yes	7.5	High quality
Kim et al.; 2019	Yes	Yes	Yes	Yes	Yes	Yes	Yes	Yes	8	High quality
Ogama et al.; 2019	Yes	Yes	Yes	Yes	Yes	Yes	Yes	Yes	8	High quality
He et al.; 2020	Yes	Yes	Yes	Yes	Yes	Yes	Yes	Yes	8	High quality
Kataoka et al.; 2020	Yes	Yes	Yes	Yes	Yes	Not clear	Yes	Yes	7.5	High quality
Orlando et al.; 2020	Yes	Yes	Yes	Yes	Yes	Yes	Yes	Yes	8	High quality
Oguz et al.; 2021	Yes	Yes	Yes	Yes	Not clear	Not clear	Yes	Yes	7	High quality
Park et al.; 2021	Yes	Yes	Yes	Yes	Yes	Yes	Yes	Yes	8	High quality
Yano et al.; 2021	Yes	Yes	Yes	Yes	Yes	Yes	Yes	Yes	8	High quality
Hiromine et al.; 2022	Yes	Yes	No	Yes	Yes	Not clear	Yes	Yes	6.5	Moderate quality
Shi et al.; 2022	Yes	Yes	Yes	Yes	Yes	Yes	Yes	Yes	8	High quality
Herder et al.; 2024	Yes	Yes	Yes	Yes	Yes	Yes	Yes	Yes	8	High quality
Mooshage et al.; 2024	Yes	Not clear	Yes	Yes	No	Not applicable	Yes	Yes	5.5	Moderate quality

^*^ The assessment was according to the Joanna Briggs Institute (JBI) Critical Appraisal Checklist for analytical cross-sectional studies

• Item 1 Were the criteria for inclusion in the sample clearly defined?

• Item 2 Were the study subjects and the setting described in detail?

• Item 3 Was the exposure measured in a valid and reliable way?

• Item 4 Were objective, standard criteria used for measurement of the condition?

• Item 5 Were confounding factors identified?

• Item 6 Were strategies to deal with confounding factors stated?

• Item 7 Were the outcomes measured in a valid and reliable way?

• Item 8 Was appropriate statistical analysis used?

**Table 4:** Quality assessment of the methodologies of included cohort studies

	Sugimoto et al.; 2021	Sencan et al.; 2022
Were the two groups similar and recruited from the same population?	Yes	Yes
Were the exposures measured similarly to assign people to both exposed and unexposed groups?	Yes	Yes
Was the exposure measured in a valid and reliable way?	Yes	Yes
Were confounding factors identified?	Yes	No
Were strategies to deal with confounding factors stated?	Unclear	Not applicable
Were the groups/participants free of the outcome at the start of the study (or at the moment of exposure)?	No	Yes
Were the outcomes measured in a valid and reliable way?	Yes	Yes
Was the follow up time reported and sufficient to be long enough for outcomes to occur?	Yes	Yes
Was follow up complete, and if not, were the reasons to loss to follow up described and explored?	Yes	Yes
Were strategies to address incomplete follow up utilized?	Unclear	Unclear
Was appropriate statistical analysis used?	Yes	Yes
**Total score**	9	8.5
**Level of quality**	High quality	Moderate quality

^*^ The assessment was according to the Joanna Briggs Institute (JBI) Critical Appraisal Checklist for cohort studies. Please note, the secondary data analysis of the cohort study (Terada et al.; USA; 2022) was not included as that study did not report primary data

### Characteristics of the Studies

Table 2 presents the characteristics of the included studies. Of the 17 studies, 14 articles were cross-sectional [26–36, 38, 39, 41, 42], two articles reported a cohort study [37, 39], and one study [40] reported the secondary data analysis results. According to the study selection criteria above, no meta-analysis is required for this systematic review.

Five studies were conducted in Japan [29, 30, 33, 39, 41], three in Korea [31, 36, 42], two in China [27, 38], two in Turkey [34, 37], two in Germany [28, 32], and the others in Singapore [26], Italy [35], and USA [40] each. There were 8,392 adults with diabetes included in this review, and one study included only males [42]. The mean age of participants included in this review was reported in 16 studies [26, 27, 29–42], ranging from 52 to 75 years old. According to the body mass index [43], three studies included participants who were obese [31, 34, 40].

In addition, 14 articles specified the type of diabetes, with 12 studies including T2D only [26, 27, 30, 32–38, 40, 41] and two studies including both type 1 diabetes (T1D) and T2D [29, 39]. Five studies examined diabetes multimorbidity, including cognitive impairment, diabetic peripheral neuropathy, cardiovascular disease, retinopathy, and diabetic polyneuropathy [30, 33, 35, 36, 41].

### Measurements for muscle functions and glycemic variability

The muscle function measurements varied across the included studies. The diagnosis of sarcopenia was reported in eight studies, with three studies using the European Working Group on Sarcopenia in Older People (EWGSOP) criteria [27, 34, 37] and five studies using the Asian Working Group for Sarcopenia (AWGS) criteria [26, 29, 33, 38, 39]. Two studies further specified the definition of probable sarcopenia [27, 37]. Five studies examined the gait test to assess the physical performance of participants, including the 4-m gait test [29, 37, 39] and 6-m gait speed [26, 34]. Nine studies examined the handgrip strength test to assess the muscle strength of participants [26, 27, 29, 33, 34, 36, 37, 39, 42]. The bioelectrical impedance analysis (BIA) [26–30, 34, 37, 39] and dual energy X-ray absorptiometry (DXA) scan [31, 33, 36, 38, 40, 41] were used in 14 studies to assess the body muscle mass of participants. One study [35] used the maximal voluntary contraction and endurance time to assess muscle fatigability. Additionally, one study [32] calculated the constant of the musculature’s microvascular permeability, extravascular extracellular volume fraction, and plasma volume fraction to assess the functionality and structural integrity of muscles.

The devices for BIA and the handgrip strength test provided in the articles also varied, with only three studies [29, 37, 39] using the same device for BIA and two studies [29, 39] for the handgrip strength. Except for two studies [33, 41] that did not provide the device information, the other studies [31, 36, 38, 40, 42] used the same device for DXA—Hologic Discovery A.

Similarly, different glycemic variability metrics were examined in the included studies. The fasting plasma glucose level and the HbA1c level were the metrics representing insulin resistance in five studies [27, 30, 33, 35, 37]. Another five studies used only HbA1c as the metric [26, 29, 34, 39, 40]. Two studies used three metrics including the fasting plasma glucose level, HbA1c, and the homeostasis model assessment of insulin resistance (HOMA-IR) [28, 36]. The other studies used either fasting plasma glucose level and HOMA-IR [32], HbA1c and HOMA-IR [31], fasting plasma glucose level, fasting insulin level, and HbA1c [41], fasting plasma glucose level, fasting insulin level, HbA1c, and HOMA-IR [42], or the largest amplitude of glycemic excursions and standard deviation of blood glucose to calculate glucose fluctuation [38].

### Relationship between muscle dysfunctions and glycemic variability

Eight studies focused on the relationships between various types of muscle dysfunction measurements and glycemic variability among adults with diabetes only. However, the research findings were not consistent. Of these, four studies found no statistically significant associations between the HbA1c level and muscle dysfunction parameters (including sarcopenia, skeletal muscle mass index (SMI), gait speed, handgrip strength, and muscle quality) [26, 29, 39, 42]. It is worth noting that, when participants were divided into groups based on their HbA1c values, those with a HbA1c level of ≥ 8.5% showed significantly decreased muscle quality [42]. SMI as well as gait speed were significantly increased when there was a decrease in HbA1c value by ≤ 1.0% [39]. The other four studies reported a significant association between the glycemic variability metrics and muscle strength and used a different cut-off value of the HbA1c level [27, 33] and muscle mass [28, 38]. Increased HbA1c and fasting glucose levels were significantly associated with low muscle strength in adults with diabetes, in particular among those with HbA1c > 7%.

Three studies reported the associations between glycemic control and sarcopenic obesity (SO) among adults with diabetes [31, 34, 40]. Two of these studies used the fat mass/fat-free mass ratio to define SO and as a criterion to divide participants into different groups [34, 40]. They both showed a positive correlation between fat mass/fat-free mass and HbA1c level and indicated poor glycemic control was an independent risk factor for SO. Terada et al. (2022) also reported their findings stratified by participants’ sex. Compared to males, managing the HbA1c level is more important for females with diabetes and low fat and low muscle mass. Kim et al. (2019) reported that adults with newly diagnosed and drug-naïve T2D had a higher risk for insulin resistance if they had SO than adults without SO.

Five studies focused on the relationship between glycemic management and muscle dysfunction in adults with diabetes complications. The research outcomes, diabetes types, and muscle dysfunction parameters were varied, and the research findings were not consistent. One study [35] found that the change in muscle fatigability was not statistically associated with the HbA1c level, while another study [41] demonstrated an association between muscle mass and the fasting plasma insulin level in non-obese heart failure adults with T2D. One study [36] reported that, among adults with diabetes and higher HbA1c [≥ 7.1%], the combination of low muscle mass and low handgrip strength was significantly associated with T2D and cardiovascular diseases. A further study [30] found that the HbA1c level was negatively associated with muscle mass in adults with T2D and diabetic polyneuropathy. Another study [33] conducted among adults with diabetes and cognitive impairment found that participants with sarcopenia had larger glucose level fluctuations than non-sarcopenia participants.

## Discussion

This systematic review aimed to explore the relationship between muscle dysfunction and diabetes in adults, with a particular focus on how glycemic variability impacts muscle health and the development of diabetes-related complications. The review included 17 studies, predominantly cross-sectional, conducted in various countries with a total of 8,392 participants. While there was some inconsistency, the findings identified poor glycemic control as an independent risk factor for sarcopenic obesity and highlighted the association between higher HbA1c levels and lower muscle mass and strength in adults with diabetes-related complications.

The majority of included studies were conducted in Asian countries. It is not surprising because Asian countries (e.g., India, China, and Japan) accounted for the largest number of people with diabetes globally [44]. It is important to note that Asian people who are living with type 2 diabetes always have a lower mean body mass index and higher insulin resistance compared to other ethnic groups [45, 46]. In addition, the Asian population with diabetes take much more carbohydrates daily than the Western population [47]. Therefore, the results reported in this review may not be generalisable to all people with diabetes worldwide and more research is required from other continents regarding the relationship between muscle dysfunction and diabetes.

The relationship between glycemic variability and muscle dysfunction in older adults with diabetes was inconsistent across the studies. Several factors may contribute to these discrepancies. Firstly, the included studies varied significantly in their design, sample size, and population characteristics. Differences in age, sex, ethnicity, and diabetes duration among participants could influence the observed relationship between glycemic variability and muscle dysfunction [19]. Additionally, the use of different methods to assess muscle function (e.g., handgrip strength, gait speed, BIA, DXA) and glycemic variability (e.g., HbA1c, fasting plasma glucose, HOMA-IR) can result in varying outcomes. The lack of standardized measurement tools across studies makes it challenging to compare results directly [21]. Furthermore, the presence of confounding factors such as physical activity levels, nutritional status, and comorbidities (e.g., cardiovascular diseases, neuropathy) can affect the observed relationship between glycemic variability and muscle dysfunction. Studies that do not adequately control for these factors may report inconsistent findings [17].

Poor glycemic management was identified as an independent risk factor for sarcopenic obesity. This finding is supported by the understanding that poor glycemic control is often associated with insulin resistance, which can impair muscle protein synthesis. In addition to stimulating glucose uptake and metabolism, insulin is an anabolic hormone that promotes muscle growth, and resistance to its effects can lead to muscle atrophy and reduced muscle mass [19]. Additionally, hyperglycemia and poor glycemic control can lead to chronic low-grade inflammation, which is known to contribute to muscle wasting and sarcopenia [48]. Inflammatory cytokines such as TNF-α and IL-6 can promote muscle protein breakdown and inhibit muscle regeneration [49]. Poor glycemic control can also exacerbate adipose tissue dysfunction, leading to increased fat infiltration in muscle (myosteatosis) [50]. This can impair muscle function and contribute to the development of sarcopenic obesity, where both muscle loss and fat gain occur simultaneously [21].

Higher HbA1c levels were associated with lower muscle mass and strength in older adults with diabetes-related complications. Chronic hyperglycemia can increase oxidative stress and mitochondrial dysfunction in muscle cells, leading to impaired muscle function and reduced muscle mass over time [51]. Diabetes-related complications such as diabetic neuropathy and microvascular disease can impair blood flow to muscle, leading to muscle ischemia and atrophy. Poor glycemic management can exacerbate these complications, further reducing muscle mass and strength [52]. Additionally, adults with poorly managed diabetes may experience nutritional deficiencies due to malabsorption or dietary restrictions, which can affect muscle health, as adequate nutrition is essential for maintaining muscle mass and function [53].

Our review also highlights the complex relationship between glycemic variability and muscle dysfunction, emphasising the need to consider multiple influencing factors. Multimorbidity is common in older adults with T2D, and five out of the 17 studies reported relevant results. These comorbidities exacerbate muscle dysfunction and complicate diabetes management [20]. For example, cardiovascular disease limits exercise capacity [54]; diabetic neuropathy causes muscle atrophy [55]; and cognitive impairment hinders effective diabetes management [56], leading to poor glycemic control and further muscle deterioration. Polypharmacy is often necessary to manage comorbidities but can adversely affect muscle health. Statins, a class of cholesterol-lowering medications commonly prescribed for cardiovascular disease, are associated with adverse effects such as myalgia, myopathy and rhabdomyolysis, which can interfere with muscle cell metabolism and function [57–59]. These side effects can further impair physical function in older adults with T2D. Obesity is a known risk factor for reduced muscle volume and physical activity and three studies reported related results. Obesity can lead to sarcopenic obesity, significantly impairing mobility and increasing the risk of falls and fractures. Obesity-related inflammation and insulin resistance also further deteriorate muscle quality and function [60, 61]. Therefore, multimorbidity, polypharmacy, and obesity act as confounding factors in the relationship between muscle quality and glycemic variability.

Our review has several strengths. It provides a comprehensive overview of the current literature on the relationship between glycemic variability, muscle dysfunction, obesity, and diabetes-related complications in older adults. The use of the PRISMA guidelines ensures a rigorous and transparent review process. The inclusion of studies with diverse populations and settings enhances the generalizability of the findings.

While our review provides valuable insights, there are several limitations to consider. First, the review only included peer-reviewed journal articles written in English, which may introduce language bias. Second, children and adolescents with diabetes were excluded from the literature search, which may introduce selection bias and limit the applicability of the findings to older adults. Third, most studies included in this review were undertaken in Asian countries, which may lead to geographic bias and more research in this field is required focusing on people living with diabetes on other continents. Additionally, the literature search was conducted in three major medical and health electronic databases, excluding grey literature, which may have resulted in publication bias.

The findings of our review underscore the need for tailored interventions to improve muscle health and manage obesity and diabetes in older adults. Interventions should focus on improving glycemic control to mitigate the risk of sarcopenic obesity and related complications. Healthcare professionals should adopt comprehensive management strategies that address both glycemic management and muscle health. These strategies may include personalized exercise programs, nutritional support, and regular monitoring of glycemic variability and muscle function. Tailored interventions that address these factors simultaneously are crucial for improving muscle health and overall quality of life in this population.

Future research should include longitudinal studies to better understand the causal relationships between glycemic variability, muscle dysfunction, and diabetes-related complications. Long-term studies can provide insights into the progression of sarcopenic obesity and the effectiveness of interventions over time. More studies focusing on diverse populations, including different ethnic and socio-economic groups, are needed to develop effective, culturally sensitive interventions. Research should also explore the impact of socio-economic factors, access to healthcare, and cultural beliefs on the management of muscle health, obesity, and diabetes. Future studies should aim to standardize the measurements of muscle function and glycemic variability to facilitate comparison and synthesis of findings. Consistent use of validated tools and criteria will enhance the reliability and validity of research outcomes.

## Conclusion

This systematic review presents the complex and inconsistent relationship between glycemic variability and muscle dysfunction in older adults with diabetes. Poor glycemic control has emerged as a significant risk factor for sarcopenic obesity, emphasizing the need for optimal glycemic management to prevent muscle deterioration and associated complications. The findings highlight the necessity for tailored interventions that address both glycemic control and muscle health. Future research should focus on longitudinal studies and standardized measurement methods to better understand these relationships and develop effective, culturally sensitive interventions for older adults with diabetes. Comprehensive management strategies, including personalized exercise programs, nutritional support and regular glucose monitoring, are essential to improve clinical outcomes in this population.

## Data Availability

No datasets were generated or analysed during the current study.

## Abbreviations

AIHW: Australian Institute of Health and Wellbeing
AWGS: Asian Working Group for Sarcopenia
BIA: Bioelectrical impedance analysis
DXA: Dual energy X-ray absorptiometry
EWGSOP: European Working Group on Sarcopenia in Older People
HbA1c: Glycated hemoglobin A1c
HOMA-IR: Homeostasis model assessment of insulin resistance
JBI: Joanna Briggs Institute
PRISMA: Preferred Reporting Items for Systematic Reviews and Meta-Analyses
SMI: Skeletal muscle mass index
SO: Sarcopenic obesity
T1D: Type 1 diabetes
T2D: Type 2 diabetes
WHO: World Health Organisation
